# The Dental Colleges

**Published:** 1888-11

**Authors:** 


### The Dental Colleges.

In all the dental colleges, from which we have heard, the classes are larger than ever before. This, in a certain sense, is gratifying; and especially if there is a better class of students seeking to enter the profession through this channel. It is to be hoped that the day is near at hand, if not already here, that there will no longer be ground for the oft repeated saying, that "if a young man is a failure at everything else he could take up dentistry.

The time is certainly at hand when college faculties should look somewhat at least, to the qualifications, both natural and acquired, of those who apply for entrance to their classes.

The day has passed when any faculty can say that it has no control over this matter. More than hitherto colleges will be held responsible for failure to make a reasonable discrimination in this respect.

